# Prevalence and predictors of heart failure among patients on maintenance hemodialysis therapy at Muhimbili National Hospital in Tanzania: a cross-sectional study

**DOI:** 10.1186/s43044-021-00223-z

**Published:** 2021-10-30

**Authors:** Puneet Kishore Bramania, Paschal Joseph Ruggajo, Francis Fredrick Furia

**Affiliations:** 1 Department of Internal Medicine, Muhimbili University of Health and Allied Sciences, P.O.Box 65001, Dar es Salaam, United Republic of Tanzania; 2 Department of Pediatrics and Child Health, Muhimbili University of Health and Allied Sciences, Dar es Salaam, United Republic of Tanzania

**Keywords:** Heart failure, Hemodialysis patients, Tanzania

## Abstract

**Background:**

Heart failure among patients on hemodialysis therapy portends poor outcomes. Traditional risk factors like aging, hypertension and diabetes mellitus are relatively common in these patients and may not accurately predict the occurrence of heart failure. Such patients may have other factors that contribute to heart failure. This study aimed to investigate the prevalence and predictors of heart failure among patients on maintenance hemodialysis at Muhimbili National Hospital in Dar es Salaam, Tanzania.

**Results:**

Among 160 patients on maintenance hemodialysis, 49 (30.6%) were female. The mean age of patients was 52.2 ± 13.3 years. Almost all patients had hypertension and 69 (43.1%) had diabetes mellitus. Heart failure was prevalent in 17 (10.6%) patients. On multivariate analysis, presence of angina, intradialytic hypertension, and anemia were independent predictors of heart failure. Patients with heart failure had significantly higher malnutrition inflammation scores and erythropoietin resistance indexes.

**Conclusions:**

Heart failure among hemodialysis patients correlates with the presence of angina, intradialytic hypertension, and anemia. Patients with heart failure had a greater degree of malnutrition–inflammation complex, and erythropoietin resistance. Patients with these conditions require a thorough cardiac evaluation and appropriate treatment.

## Background

Cardiovascular disease (CVD) is accompanied by high morbidity and mortality among patients with chronic kidney disease (CKD) [1, 2]. In CKD left ventricular hypertrophy is rampant and is accompanied by high mortality [3]. Patients on hemodialysis (HD) further succumb to pathological cardiovascular impairment attributed to chronic inflammation, anemia, vascular calcifications, and recurrent fluid volume shifts [2]. In a study done among CKD patients at Muhimbili National Hospital (MNH) in Tanzania, heart failure (HF) was prevalent in 27.7% and was a significant predictor of left ventricular systolic dysfunction [4]. Congestive heart failure was noted in 36% of incident HD patients in a study done in the USA [5]. Traditional risk factors like aging, hypertension, diabetes mellitus and measures of atherosclerosis were associated with heart failure in these patients [5].

**Fig. 1:**
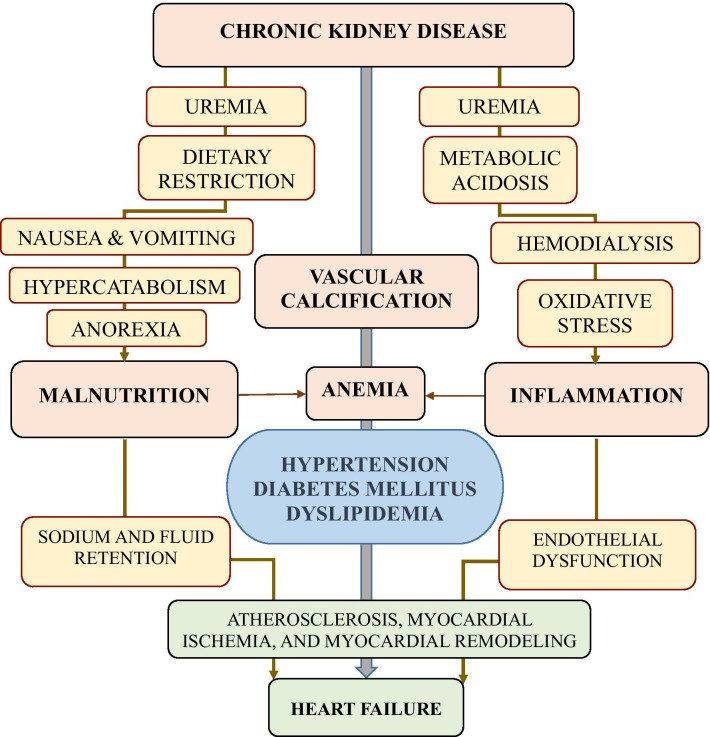
A conceptual framework to show the link between chronic kidney disease and its related factors, co-morbidities, and the occurrence of heart failure

Additionally, the malnutrition inflammation complex syndrome (MICS) in HD patients is accompanied by progressive atherosclerosis [6, 7]. Myocardial ischemia subsequently results in myocardial apoptosis and fibrosis, thus minimizing the functional cardiac reserve [2]. Moreover, MICS is linked with inter-dialytic fluid retention that can aggravate heart failure [8]. The link between CKD-related factors, co-morbid conditions, and the occurrence of heart failure is illustrated in Fig. 1. There is a paucity of information on predictors of heart failure among dialysis patients in our setting. Therefore, we aimed to investigate the prevalence and predictors of heart failure among patients on maintenance hemodialysis at Muhimbili National Hospital in Dar es Salaam, Tanzania.

## Methods

### Study design and settings

A hospital-based cross-sectional study was conducted in two hemodialysis centers under the Muhimbili National Hospital in Dar es Salaam, Tanzania, from September to November 2019.

### Study population and sample size

This study included incident hemodialysis patients aged above 18 years and on dialysis therapy for at least 3 months. We excluded patients with severe respiratory distress and those who were mentally incapacitated. This study included 160 hemodialysis patients who were part of the study done to determine the prevalence of MICS among patients on maintenance hemodialysis at Muhimbili National Hospital in Dar es Salaam, Tanzania [9].

### Data collection methods

Patients’ demographic, dialysis-related, and other clinical information [presence of diabetes, hyperlipidemia, human immunodeficiency virus (HIV) disease, angina, and symptoms of heart failure] were collected using questionnaires. Their heights were measured using a stadiometer and post-dialysis weight (dry-weight) determined using a standard weighing scale. The total monthly dose of erythropoietin (EPO) over the preceding month was obtained from their dialysis records. Likewise, dry weight over the past three months was also obtained from their records. Blood pressures were measured before and after the dialysis procedure on the day of data collection.

### Laboratory tests

Patients’ blood specimens were drawn before and after the hemodialysis procedure and were tested for pre-dialysis complete blood count (CBC), albumin, total cholesterol, transferrin, ferritin, C-reactive protein (CRP), and urea. The post-dialysis urea was also measured. The machines used for analyzing these tests included CELL DYN 3700 for CBC, COBAS INTEGRA 400 for CRP level, and ARCHITECT PLUS for all other biochemical tests.

### Study variables

The main outcome variable, heart failure was defined based on symptoms according to the Framingham criteria [10] that is having both orthopnea and paroxysmal nocturnal dyspnea (major criteria) or either of these with at least two of the symptoms in minor criteria (ankle edema, dyspnea on exertion, and nocturnal cough). Hypertension was defined as having systolic blood pressure (SBP) above 140 mmHg and or diastolic blood pressure (DBP) above 90 mmHg [11]. Intradialytic hypertension was defined as a rise in mean arterial pressure > 15 mmHg within or immediately post-dialysis [12]. The malnutrition inflammation score (MIS) was assessed using 10 components: (1) weight change compared to three months ago, (2) dietary intake, (3) gastrointestinal symptoms, (4) nutritional-related functional impairment, (5) comorbidity status, (6) extent of fat loss, (7) extent of muscle loss, (8) body mass index, (9) serum albumin level, and (10) serum transferrin level. Each of them graded from 0 (normal) to 3 (severe). MICS was defined as having MIS of 6 or above [9]. Dialysis adequacy was assessed using the Urea Reduction Ratio (URR) calculated as (Pre-dialysis urea − Post-dialysis urea) ÷ Pre-dialysis urea × 100%. A URR of at least 65% was considered as adequate dialysis [13]. Erythropoietin resistance index (ERI in units/kg per g/dl) calculated as weekly EPO dose (units) ÷ hemoglobin (g/dl) ÷ dry-weight (kg) [14].

### Data management and analysis

Questionnaires were reviewed for completeness followed by data entry into the statistical package of social sciences (SPSS) software version 20 that was used for data analysis. Categorical variables were analyzed using the proportions and compared with the outcome, heart failure using the Chi-square test. Continuous variables were analyzed using mean and median that were compared with the outcome of heart failure using Analysis of variance (ANOVA) and Mann–Whitney U-test, respectively. The binary logistic regression analysis was utilized to determine the odds of heart failure. The variables with a univariate *p* < 0.2 were included in the multivariate analysis, a *p* value of < 0.05 was considered statistically significant.

### Ethical considerations

Ethical approval was granted by the Institutional Review Board of Muhimbili University of Health and Allied Sciences (MUHAS). All patients provided written informed consent.

## Results

### Socio-demographic and clinical characteristics of the study population

Among the 160 patients on maintenance hemodialysis, 49 (30.6%) were female, and one-third were above 60 years of age. The mean age of patients was 52.2 ± 13.3 years. Almost two-thirds, 106 (66.3%) were on HD for at least 1 year and the overall median duration on HD was 18 (8.25–29.75) months. Diabetes mellitus was present in 69 (43.1%) patients. Nine (5.6%) patients reported being diagnosed with hyperlipidemia. HIV disease was present in 15 (9.4%) patients whereas 9 (5.6%) patients had hepatitis B infection. Most patients, 154 (96.3%) reported being diagnosed to have hypertension, among these 140 (90.9%) were using anti-hypertensive medications at the time of the study. The classes of anti-hypertensive medications used could be enquired in 104 patients: Calcium channel blockers were the commonest used anti-hypertensive drugs 82 (78.8%) followed by hydralazine 63 (60.6%), beta-blockers 35 (33.7%), and angiotensin-converting enzyme inhibitors or angiotensin receptor blockers 9 (8.7%).

The mean pre-dialysis SBP and DBP were 156 ± 22 mmHg and 82 ± 13 mmHg, respectively. The mean post-dialysis SBP and DBP were 153 ± 23 mmHg and 83 ± 12 mmHg, respectively. Pre-dialysis systolic hypertension (> 140 mmHg) was present in 78.8% and pre-dialysis diastolic hypertension (> 90 mmHg) was present in 23.1%. Intradialytic hypertension defined as a rise of mean arterial pressure above 15 mmHg was prevalent in 9 (5.6%) and 23 (14.4%) patients who had a post-dialysis SBP rise of above 10 mmHg.

The mean hemoglobin of the study population was 9.3 ± 1.9 g/dl, and 131 (82%) had hemoglobin below 11 g/dl. The mean URR was 70.2 ± 11.3%, and 122 (76.3%) were receiving adequate HD as per National Kidney Foundation Disease Outcomes Quality Initiative (KDOQI) recommended target URR of at least 65% [13]. The malnutrition inflammation complex syndrome was prevalent in 74 (46.3%) patients, the MIS ranged from 0 to 28 with mean (± SD) and median value of 7.6 (± 5.1) and 5, respectively.

### Prevalence of heart failure among patients on maintenance hemodialysis

**Fig. 2:**
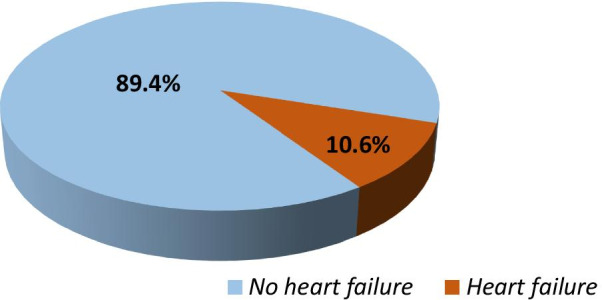
Pie-chart to show the prevalence of heart failure among patients on maintenance hemodialysis at Muhimbili National Hospital in Tanzania

Of the 160 patients on HD, 17 (10.6%) had heart failure based on the Framingham criteria (Fig. 2).

Exertional fatigue was very common, present in 127 (79.4%) patients. Exertional dyspnea, orthopnea, paroxysmal nocturnal dyspnea, nocturnal cough was present in 38.8%, 16.3%, 5.6% and 8.1%, respectively. On clinical examination, 43 (26.9%) were assessed to have bilateral ankle pitting edema. Eight (5%) patients reported having chest pain on exertion (angina).

### Predictors and factors associated with heart failure among patients on maintenance hemodialysis

On univariate binary logistic analysis advanced age, presence of diabetes, angina, pre-, and post-dialysis hypertension, intradialytic hypertension, and higher MIS resulted in higher odds of HF. Hemoglobin levels negatively correlated with the occurrence of HF. On a multivariate logistic analysis intradialytic hypertension, the presence of angina, and lower hemoglobin (anemia) were independent predictors of heart failure (Table 1).

**Table 1:** Predictors of heart failure among patients on hemodialysis therapy

Characteristic	Unadjusted odds ratio (95% CI)	*p* Value	Adjusted odds ratio (95% CI)	*p* Value
Age of patient (years)	1.03 (0.99–1.07)	0.20	1.03 (0.97–1.08)	0.34
Male gender	1.07 (0.35–3.21)	0.91	–	–
Diabetes mellitus	2.03 (0.73–5.65)	0.17	1.83 (0.50–6.70)	0.36
Hyperlipidemia	1.06 (0.12–9.0)	0.96	–	–
Angina	10.7 (2.39–47.8)	< 0.05	5.91 (1.06–33.0)	< 0.05
Pre-dialysis hypertension	4.43 (0.57–34.7)	0.16	6.66 (0.28–161)	0.24
Post-dialysis hypertension	3.23 (0.71–14.7)	0.13	1.82 (0.27–12.5)	0.54
Intra-dialytic hypertension	4.89 (1.10–21.7)	< 0.05	7.71 (1.28–46.0)	< 0.05
HIV Disease	0.58 (0.07–4.68)	0.61	–	–
HD duration (hours)	1.01 (0.98–1.03)	0.57	–	–
HD frequency (twice/week)	1.01 (0.27–3.78)	0.99	–	–
Urea reduction ratio (%)	1.02 (0.97–1.06)	0.55	–	–
Body mass index (kg/m^2^)	0.96 (0.84–1.10)	0.53	–	–
Serum albumin (g/dl)	0.89 (0.32–2.49)	0.83	–	–
Hemoglobin (g/dl)	0.72 (0.54–0.96)	< 0.05	0.70 (0.48–0.99)	< 0.05
Malnutrition Inflammation Score	1.10 (1.01–1.19)	< 0.05	1.03 (0.93–1.14)	0.61

Patients with HF had significantly lower mean hemoglobin (8.3 vs. 9.4 g/dl, *p* < 0.05). The median MIS was significantly higher among patients with HF compared to those without HF (10 vs. 5, respectively, *p* = 0.04).

Patients with MICS had a higher prevalence of HF however, this was not statistically significant (14.9% vs. 7%, *p* = 0.11). Erythropoietin resistance index (ERI) as defined by weight-adjusted weekly erythropoietin dose (Units) per dry weight (kg) per hemoglobin (g/dl) was significantly higher in patients with heart failure (Mean ERI 25.4 vs. 19.3 Units/kg per g/dl, *p* < 0.05) (Table 2).

**Table 2:** Factors associated with heart failure among patients on hemodialysis therapy

Characteristic	*N*	Heart failure		*p* Value
		Present	Absent	
Age (years)	160	56.1 ± 11.4	51.8 ± 13.4	NS
% Male	160	70.6	69.2	NS
% Diabetes mellitus	160	58.8	41.3	NS
Pre-dialysis MAP (mmHg)	160	105 ± 12	107 ± 15	NS
Post-dialysis MAP (mmHg)	160	110 ± 10	106 ± 14	NS
Duration on HD (months) *	160	18 (9.5–39)	18 (7–28)	NS
Urea reduction ratio (%)	160	71.8 ± 7.9	70.0 ± 11.7	NS
Body mass index (kg/m^2^)	160	21.9 ± 3.7	22.5 ± 4.0	NS
Serum albumin (g/dl)	160	3.69 ± 0.41	3.72 ± 0.49	NS
Total cholesterol (mg/dl)	160	138 ± 41	154 ± 39	NS
C-reactive protein (mg/l) *	103	22.4 (11.2–62)	15 (6.0–34)	NS
Malnutrition inflammation score (MIS) *	160	10 (5–12.5)	5 (4–10)	0.04
Total leucocyte count (× 10^9^/l)	160	4.7 ± 1.8	5.1 ± 2.3	NS
Hemoglobin (g/dl)	160	8.3 ± 2.2	9.4 ± 1.9	0.02
Serum transferrin (mg/dl)	160	171 ± 32	196 ± 51	NS
Serum ferritin (ng/ml)*	102	115 (74–243)	117 (54–275)	NS
Erythropoietin resistance index, ERI (U/kg per g/dl)	120	25.4 ± 10.8	19.3 ± 8.0	0.01

*Median (Interquartile range), *NS* not significant (*p* ≥ 0.05)

## Discussion

In this study, we evaluated 160 patients on maintenance hemodialysis at MNH in Dar es Salaam, Tanzania to determine the prevalence of heart failure. Heart failure was noted in 10.6% of the patients. Anemia, the presence of angina, and intradialytic hypertension were independent predictors of HF. Compared to those without, patients with HF had a higher median malnutrition inflammation score, and mean erythropoietin resistance index. A lower prevalence of HF was found in our study when compared to an earlier study done at MNH [4]. This may be explained by better fluid volume control as depicted by a high proportion (76.3%) of HD patients in our study receiving adequate dialysis [13]. Excess fluid (preload) increases the cardiac load that stimulates the renin–angiotensin–aldosterone system (RAAS) subsequently resulting in left ventricular (LV) remodeling [2, 8, 12]. Sympathetic over-stimulation from anemia similarly induces LV hypertrophy that causes diastolic dysfunction [2, 4]. Treatment of anemia using erythropoietin stimulating agents is the mainstay. However, some patients may not respond well especially if concomitant iron deficiency and chronic inflammation are not addressed [14, 15].

Patients with HF were noted to have significantly higher Erythropoietin resistance. The latter has been linked with higher mortality [15]. Erythropoietin-resistant anemia will necessitate higher EPO doses if underlying causes are not adequately treated [14, 16]. Higher EPO doses result in high blood viscosity and elevated blood pressures that increase afterload which can precipitate HF [16].

The presence of HF is significantly associated with having higher MIS. This may be attributed to greater inter-dialytic fluid retention that occurs in patients with MICS [8]. Ongoing weight losses in patients with MICS may limit adequate fluid control. Vlatković et al. found that patients with low BMI are at higher risk of fluid overload [8]. Congestive heart failure is accompanied by increased cytokines like TNF-alpha that propels cardiac cachexia [17]. In addition, MICS is accompanied by hypoalbuminemia that promotes fluid shifts from the intravascular compartment [6–8].

High inter-dialytic weight gain is postulated to result in intra-dialytic hypertension [12, 18]. In our study, intra-dialytic hypertension significantly correlated with the occurrence of heart failure. Intra-dialytic hypertension has also been linked with sodium retention, endothelial dysfunction, RAAS, and sympathetic over-stimulation. All these factors propagate LV remodeling increasing the risk of heart failure [12, 18].

Malnutrition and inflammation augment atherosclerosis and is therefore interrelated as Malnutrition–inflammation–atherosclerosis (MIA) syndrome which is linked to poor cardiovascular outcomes [7]. Coronary atherosclerosis in HD patients is widespread and results in gradual myocardial loss causing systolic dysfunction [2, 7]. In our study patients with angina had almost six-fold higher odds of having heart failure. Hypercholesterolemia may not accurately predict coronary atherosclerosis in this population. Instead, patients with low cholesterol levels have advanced CVD. In this study patients with HF had lower cholesterol levels. This is consistent with the concept of reverse epidemiology which is mostly attributed to chronic inflammation, oxidative stress, and resulting endothelial, and myocardial dysfunction [18, 19]. In patients with advanced CKD, traditional risk factors do not strongly relate to CVD unlike the general population [18, 19]. Likewise in our study aging, diabetes, hypertension, and hyperlipidemia did not significantly associate with the occurrence of HF, instead MICS correlated with HF in this population.

Management of heart failure in dialysis patients entails adequate fluid volume control, treatment of hypertension, anemia, and the use of medications like angiotensin-converting enzyme inhibitors, angiotensin receptor blockers, and beta-blockers [2]. This class of medications has been shown to reduce cardiovascular events, and mortality in hemodialysis patients [20, 21]. In view of the widespread cardiac impairment in HD patients that is certain to worsen in the setting of MICS, hypertension, and chronic anemia, it is reasonable to consider its use [2–4, 20, 21]. The limited use of these drugs by patients in our study demands a collaborative approach by nephrologists, cardiologists, and physicians in updating prescribing practices considering the CVD vulnerability of these patients.

Statins and anti-oxidants may also help curb the inflammatory syndrome that fosters atherosclerosis and myocardial damage [22, 23]. High flux hemodialysis and online hemodiafiltration allow better clearance of middle molecules implicated in inflammation, and erythropoietin refractory anemia [14].

The small number of patients and lack of echocardiography assessment were some limitations of our study. The class of heart failure was not assessed as well. The prevalence of heart failure may be under-estimated as we only included stable dialysis patients attending on an out-patient basis and excluded a few patients with severe respiratory distress.

## Conclusions

Heart failure is common among hemodialysis patients, and in this study, heart failure was found to be associated with the presence of angina, intradialytic hypertension, and anemia. Patients with heart failure had a greater degree of malnutrition–inflammation complex, and erythropoietin resistance. Such patients require a thorough cardiac evaluation, and appropriate treatment.

## Data Availability

The datasets used and/or analyzed during the current study are available from the corresponding author on a reasonable request.

## Abbreviations

ANOVA: Analysis of variance
CBC: Complete blood count
CKD: Chronic kidney disease
CRP: C-reactive protein
CVD: Cardiovascular disease
DBP: Diastolic blood pressure
EPO: Erythropoietin
ERI: Erythropoietin resistance index
HD: Hemodialysis
HF: Heart failure
HIV: Human immunodeficiency virus disease
KDOQI: Kidney foundation disease outcomes quality initiative
LV: Left ventricle
MIA: Malnutrition–inflammation–atherosclerosis
MICS: Malnutrition inflammation complex syndrome
MIS: Malnutrition inflammation score
MNH: Muhimbili National Hospital
MUHAS: Muhimbili University of Health and Allied Sciences
RAAS: Renin–angiotensin–aldosterone-system
SBP: Systolic blood pressure
SPSS: Statistical package of social welfare
URR: Urea reduction ratio
